# Internal structure of intonational categories: The (dis)appearance of a perceptual magnet effect

**DOI:** 10.3389/fpsyg.2022.911349

**Published:** 2023-01-17

**Authors:** Joe Rodd, Aoju Chen

**Affiliations:** ^1^Office of Education, Ministry of Education, Culture and Science, The Hague, Netherlands; ^2^Institute for Language Sciences, Utrecht University, Utrecht, Netherlands

**Keywords:** intonational phonology, Autosegmental-metrical theory, pitch accent, categorical perception, internal structure, perceptual magnet effect, goodness rating, differential discrimination

## Abstract

The question of whether intonation events are speech categories like phonemes and lexical tones has long been a puzzle in prosodic research. In past work, researchers have studied categoricality of pitch accents and boundary tones by examining perceptual phenomena stemming from research on phoneme categories (i.e., intonation boundary effects—peaks in discrimination sensitivity at category boundaries, perceptual magnet effects—sensitivity minima near the best exemplar or prototype of a category). Both lines of research have yielded mixed results. However, boundary effects are not necessarily related to categoricality of speech. Using improved methodology, the present study examines whether pitch accents have domain-general internal structure of categories by testing the perceptual magnet effect. Perceived goodness and discriminability of re-synthesized productions of Dutch rising pitch accent (L*H) were evaluated by native speakers of Dutch in three experiments. The variation between these stimuli was quantified using a polynomial-parametric modeling approach. A perceptual magnet effect was detected: (1) rated “goodness” decreased as acoustic-perceptual distance relative to the prototype increased (Experiment 1), and (2) equally spaced items far from the prototype were more frequently discriminated than equally spaced items in the neighborhood of the prototype (Experiment 2). These results provide first evidence for internal structure of pitch accents, similar to that found in color and phoneme categories. However, the discrimination accuracy gathered here was lower than that reported for phonemes. The discrimination advantage in the neighborhood far from the prototype disappeared when participants were tested on a very large number of stimuli (Experiment 3), similar to findings on phonemes and different from findings for lexical tones in neutral network simulations of distributional learning. These results suggest a more transient nature of the perceptual magnet effect in the perception of pitch accents and arguably weaker categoricality of pitch accents, compared to that of phonemes and in particular of lexical tones.

## Introduction

Intonational phonology concerns the mapping of phonetic-level variation in fundamental frequency (F0, also known as pitch in speech perception) to abstract units, which are then in turn mapped to meanings. The most widely accepted theory of intonational phonology, the autosegmental-metrical theory (hereafter AM theory), characterizes F0 or pitch (hereafter pitch) movement in terms of a series of high and low tones, organized sequentially (Pierrehumbert, 1980; Beckman and Pierrehumbert, 1986; Gussenhoven, 2004; Ladd, 2008; Arvaniti and Fletcher, 2020). These tones can either stand on their own as single tonal targets, or be combined into bi-tonal and tri-tonal targets. Such tonal targets can be of a lexical nature such as lexical tones in languages like Thai and Mandarin, and lexical pitch accents in languages like Tokyo Japanese and Stockholm Swedish, or of a post-lexical nature such as pitch accents in intonation languages like English, Dutch, and Italian. These tonal targets are aligned onto the segmental stream, and are organized into a phrasal structure of intermediate phrases within intonational phrases. Each type of phrase additionally potentially carries a boundary tone marking the right edge of that phrase. Just as in segmental phonology, phonetic realization rules govern the transformation of this abstract representation of the melody into a realizable pitch contour and the temporal alignment of tones to the segmental stream. However, phonetic implementation is underspecified in intonation (Arvaniti, 2011), leaving room for phonetic variation.

A critical assumption of the AM theory is that pitch accents are discrete or phonological categories, similar to lexical tones and lexical pitch accents. However, this assumption has been the subject of continuous debate in the field of prosody, because pitch accents and lexical tones are different in several aspects. First, lexical tones are far more densely distributed than pitch accents because each syllable can be specified for a lexical tone, one syllable per word is specified for a lexical pitch accent, but only some words are realized with a post-lexical pitch accent (hereafter pitch accent) in an utterance (Arvaniti and Ladd, 2009, *cf.* Xu et al., 2015). Second, pitch accents are more difficult to establish than lexical tones, because a meaning difference suffices to tell two lexical tones apart but is no sufficient to determine whether two pitch contours are from the same category or two distinct categories (Arvaniti and Fletcher, 2020). Third, the functional difference between pitch accents and lexical tones has led to the claim that lexical tones are stored in the lexicon and may thus be more consistently and precisely represented in the prosodic system than pitch accents are (Hallé et al., 2004; Francis et al., 2008). Finally and probably most importantly, there is still no consensus on what should be taken as empirical evidence for or against the categoricality of pitch accents (Gussenhoven, 1999; Prieto, 2012).

The present study aims to contribute to a clearer understanding of categoricality of pitch accents from an understudied perspective. Because this line of research is deeply rooted in the methodology used in research on categoricality of phoneme categories, we will first briefly review two perceptual phenomena that are tested to support the categoricality of phonemes (“Categoricality of phoneme categories”), then offer a brief critical review of past research on categoricality of pitch accents following the methodology stemming from research on phoneme categories (“Past work on categoricality of pitch accents”), and finally outline our approach to categoricality of pitch accents and present our hypotheses and predictions (Section The current study).

### Categoricality of phoneme categories

Categoricality of phoneme categories has been experimentally studied by testing two perceptual phenomena, i.e., discrimination sensitivity peaks at phonemic boundaries and poor discrimination sensitivity within phonemic boundaries, reaching minima near the best exemplars or prototype of a category. The former is known as categorical perception, typically established through the so-called categorical perception (CP) paradigm consisting of an identification task and a discrimination task (Liberman et al., 1957). However, this term is also strongly associated with a class of hypothesized mechanisms, which assume that phonemes are perceived in terms of phonemic categorization or phonemic labelling (see Iverson and Kuhl, 2000 for a brief review). To separate the perceptual phenomenon and its hypothesized mechanisms, we will use the term *phoneme boundary effect* (Wood, 1976) in this paper, following Iverson and Kuhl (2000). The phenomenon of minimum discrimination near the prototype of a phoneme category is known as the *perceptual magnet effect* (Kuhl, 1991; Davis and Kuhl 1994), established through perceptual goodness rating and discrimination tasks. These two perceptual effects appear to stem from different processes. More specifically, Iverson and Kuhl (2000) tested the perception of English /i/ and /e/ vowels in conditions differing in the range of stimuli presented in each block of stimuli (or context variance, following Macmillan et al., 1988). They found that in the condition with reduced context variance, the sensitivity peaks near vowel boundaries disappeared whereas the sensitivity minima remained. This finding was interpreted to mean that the phoneme boundary effect may arise from cognitive encoding strategies such as perceptual anchoring (Macmillan et al., 1988), but the perceptual magnet effect from auditory processing (Iverson and Kuhl, 2000).

Animal studies (e.g., Kuhl and Miller, 1975; Kuhl et al.,1978) and non-speech studies with humans (e.g., Kluender et al., 1988) have shown that the phoneme boundary effect is present in animals with no access to phonemic labels and in human listeners listening to non-speech stimuli. However, research on rhesus monkeys’ perception of vowels has yielded no evidence for a perceptual magnet effect (Kuhl, 1991). Together with Iverson and Kuhl (2000), these findings suggest a lack of a direct link between the phoneme boundary effect and the presence of phoneme categories in listeners’ mental representation. It is thus highly questionable to take evidence for a phoneme boundary effect as evidence for categoricality of phonemes.

In contrast, the presence of a perceptual magnet effect has been argued to reflect domain-general internal structure of categories (Kuhl, 1991; Lacerda, 1995). Every category has presumably an indefinite number of members or exemplars. Crucially, not all members are perceived to be good or representative exemplars of a category by listeners; members closer to best exemplars are harder to discriminate than members further away. Furthermore, the magnitude of the perceptual magnet effect in phonemic perception can be affected by individual differences in phonemic categorization and ability to label synthetic stimuli as good exemplars of phoneme categories (Iverson and Kuhl, 2000). For example, Aaltonen et al. (1997) found that listeners exhibited a perceptual magnet effect on the mismatch negativity measure only if they could consistently label their stimuli as /i/ or /y/ in Finnish. Iverson and Kuhl (2000) found that the perceptual magnet effect decreased for listeners who were less clear on which stimuli they perceived to be good exemplars of /r/ in English. Similarly, in Lively and Pisoni’s (1997) study on the perception of /i/, their listeners showed considerably more variability in goodness ratings than has been reported in other studies of the same phoneme and exhibited no perceptual magnet effect.

### Past work on categoricality of pitch accents

Over the past decades, researchers have primarily studied categoricality of pitch accents and boundary tones by examining an intonation boundary effect, the equivalent of the phoneme boundary effect, using a range of methods, such as the CP paradigm, a reaction time (RT) paradigm, and semantic identification (see Gussenhoven, 1999; Prieto, 2012; Gussenhoven and van de Ven, 2020 for reviews). Evidence for an intonation boundary effect has been at best inconsistent. For example, using the CP paradigm, Ladd and Morton (1997) examined the difference between a “normal” high and “emphatic” high pitch accent in English and found an identification boundary but no discrimination peak. When RT was measured during the identification task on a comparable stimuli set, slower reactions were found at the identification boundary, suggesting a categorical interpretation of peak height in English intonation (Chen, 2003). In Bari Italian, counter-expectational questions, narrow-and contrastive statements are all realized on L*H + L%, with varying peak heights. Savino and Grice (2011) combined the CP paradigm with the RT measurement and found that differences between the “question” meaning and either “statement” interpretation were perceived categorically, but the two “statement” meanings were not in Bari Italian. Similar findings were also reported for utterance-initial pitch peaks between (lower) statements and (higher) non-statements in Catalan (Prieto, 2004). Regarding peak alignment, Pierrehumbert and Steele (1989) used a repetition task to test for categoricality between English L* + H and L + H*. Their participants were asked to repeat stimuli from a continuum that varied in peak alignment in 20 ms steps. The repetitions fell into two categories, leading the authors to conclude that the peak alignment dimension was represented in a binary manner. However, using a CP-with-RT approach, Chen (2003) found no evidence of categorical perception on a similar stimulus continuum in British English.

However, for at least two reasons it is problematic to equate evidence for a boundary effect with evidence for the categoricality of pitch accents and conversely, to interpret a lack of evidence for a boundary effect as evidence against the categoricality of pitch accents. First, as discussed in “Categoricality of phoneme categories”, a boundary effect or categorical perception is not necessarily related to categoricality of speech categories. Second, in experiments on intonation boundary effects, meaning attributes are used as the labels to access whether two intonational events are two distinct categories in the identification task. This adaption of the CP paradigm itself raises questions on whether intonational events are stored in the mental representation as speech categories independent of meaning attributes, like phonemes and lexical tones.

Compared to research on intonation boundary effects in the perception of pitch accents and boundary tones, there is much less research on perceptual magnet effects in the perception of postulated intonational categories. But existent work has similarly yielded mixed findings. For example, Schneider and Möbius (2005) found a perceptual magnet effect for the low boundary tone (L%) but not for the high boundary tone (H%) in German when stimuli were presented as isolated sentences, but in both boundary tone categories when each stimulus was preceded by a felicitous context (Schneider et al., 2009). In both studies, the boundary tones were varied on a one-dimensional continuum of pitch height at the end of the sentence. Moreover, the prototype and non-prototype of the boundary tones were determined on a semantic basis by asking listeners to rate each stimulus on how well it represented a statement or a question, different from the approach taken in studies on phoneme categories. Adopting Schneider and co-workers’ methodology, Fivela (2012) tested for a perceptual magnet effect in H* + L and H* followed by a low phrase accent in Pisa Italian, where these accents serve a function of marking ‘continuation/reintroduction’ and ‘correction/opposition’ respectively within contrastive focus. Tokens of H* + L and H* followed by a low phrase accent were varied along a two-dimensional continuum (peak alignment, peak height). A perceptual magnet effect was found for H* + L but not for H*. In fact, for H*, discrimination was better in pairs in the vicinity of the prototype of H* than in pairs further away from it.

The perceptual magnet effect is reliant on a concept of acoustic-perceptual distance to define how far an exemplar is from the prototype of the category, and to define the spacing between pairs of items. This distance metric should be derived from quantification of the acoustic variation that causes change in category identity. In the segmental domain, this is relatively straightforward: formant frequencies characterize vowels, for instance, whilst voice onset time conveys the voicing distinction in stops. Intonation, as changes in pitch in time anchored to the segmental stream, is by definition multi-dimensional: changes in pitch scaling, peak-and valley alignment and accent duration all conceivably contribute to category identity. Furthermore, if intonational categories are like phoneme categories and have internal structure of categories, the goodness of a member as the prototype of a postulated intonational category should arguably be independent of meaning attributes. Small variation in pitch accents and boundary tones can convey subtle shades of meaning. A representative exemplar of a pitch accent to convey a certain meaning attribute may not be equally representative of that pitch accent as an abstract category in the acoustic sense. Hence, the question arises as to whether the multi-dimensional nature of intonation and the semantically-driven choice of prototypes may be the cause for absence of the perceptual magnet effect in Pisa Italian H* (Fivela, 2012) and the reliance of the perceptual magnet effect on the presence of felicitous contextual information in German boundary tones (Schneider et al., 2009).

### The current study

In the current study, circumventing methodological limitations in previous research on the perceptual magnet effect in intonational categories, we aim to find out whether pitch accents can be considered speech categories by examining whether they have domain-general internal structure of categories. To this end, we adopted parametric modeling of intonation (Reichel, 2011; Walsh et al., 2013) to quantify variation in pitch accents along five dimensions (more on this in “General methodological issues”), and tested for the presence of a perceptual magnet effect in the L*H pitch accent on the Dutch one-word utterance Mi in three experiments (“Experiment 1: Goodness rating of resynthesized stimuli”, “Experiment 2: Discrimination”, and “Experiment 3: Discrimination in a within-subject design”). The L*H pitch accent was selected because it was one of two pitch accents of which productions were systematically collected and analyzed by Chen et al. (2014). Testing L*H before the other pitch accent (i.e., H*L) is desirable because of the increased variability in shape in H*L compared to alignment in L*H found by Caspers and van Heuven (1993); if there is more variability in the shape, that implies that the perceptual space defined by CoPaSul parameters will similarly be larger, making a perceptual magnet effect easier to detect.

We hypothesize that pitch accents have internal structure, in the same way that phonemes do. If this hypothesis is true, we predict first that stimuli closer to the prototype of L*H will receive higher goodness ratings than those further away from it, and second that discrimination accuracy will be worse in stimuli closer to the prototype than in stimuli further away from it. We refer to these two predictions as the ‘gradient goodness’ symptom and the ‘differential discriminability’ symptom, respectively.

## General methodological issues

### Modeling approach

We adapted the CoPaSul (contour, parametric, and superpositional) intonation model (Reichel, 2011) to quantify pitch accent variation. CoPaSul models a linear global declination contour in the domain of the intonational phrase, then uses a series of parametrically defined third-order polynomial functions to stylize the residual movement in the domain of the accent group. We adapted CoPaSul in two ways. First, we removed the global contour, which models declination in connected speech, and was not relevant in our isolated stimuli. Second, we substituted CoPaSul’s natural polynomials for orthogonal polynomials. Natural polynomials are mathematically straightforward in computation, but the parameters are by definition correlated with each other. This is undesirable for the purposes of this study, because pairs of parameters then have a highly correlated distribution, which complicates the generation of stimuli sets that vary predictably and evenly. Using Legendre orthogonal polynomials (Grabe et al., 2007) instead of natural polynomials solves this problem without worsening the quality of stylization. This adjustment resulted in a round rather than ovoid exemplar cloud, making the calculation of acoustic-perceptual distance between exemplars and the placement of referents to test more straightforward. For convenience, we refer to the resulting model as Simplified Orthogonal CoPaSul (SOCoPaSul).

**Figure 1:**
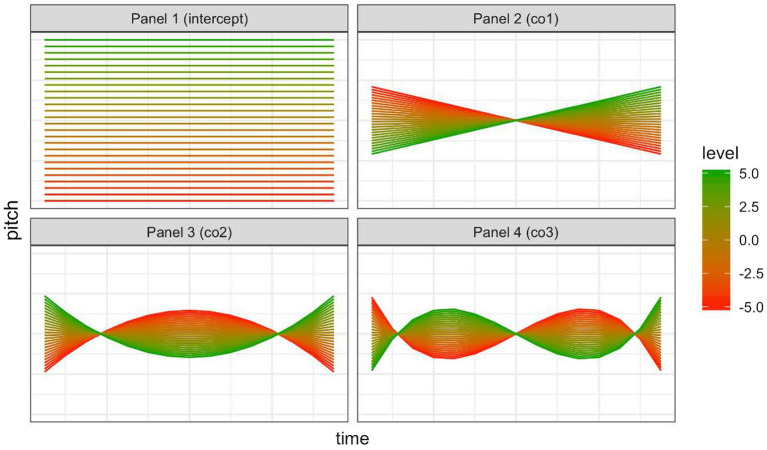
Parameters in SOCoPaSul. This figure shows, in each of the four panels, the effect on the contour shape of changing each parameter from a high value (green) to a low value (red), whilst holding the values of all the other parameters constant. Panel one depicts variation of the intercept (pitch level), panel 2 and panel 4 depicts variation in the rising or falling direction of the intonation contour and the peak alignment (CO1 and CO3), panel 3 controls peak shape, from convex to concave (CO2).

SOCoPaSul characterizes different shapes of intonation contours in terms of four parameters: a parameter controlling the local pitch level (INTERCEPT), two inter-related parameters that control the rising or falling direction of the intonation contour and the peak alignment (CO1 and CO3), a parameter controlling peak shape, from convex to concave (CO2), as shown in Figure 1. The interactions between parameter values create more complex shapes. To complete our characterization of the prosodic properties of each pitch accent exemplar, we also added its duration as a fifth metric, to capture the interaction of duration with the other parameters. The exemplars of each pitch accent were thus modeled in a five-dimensional space in SOCoPaSul. The acoustic-perceptual distance between two exemplars was the Euclidean distance in this five-dimensional space.

### The stimuli

The stimuli were generated in five steps.

#### Step 1: Selecting the prototype

We selected the prototype of L*H in Dutch using recordings from Chen et al. (2014). Adopting Caspers’s (2000) elicitation method, Chen et al. (2014) studied the realisation of L*H and H*L in Dutch and their equivalents in Mandarin Chinese, i.e., the rising and falling tones (Tone 2 and Tone 4).^1^ Caspers (2000) designed 24 ‘situational contexts’ to elicit renditions of four signal-accent intonation patterns (i.e., an accent-lending rise, an accent-lending fall, an accent-lending rise and fall on one syllable, and an accent-lending rise and a half fall on one syllable) realised on proper names in three contexts from both the ‘default’ and ‘vocative’ perspectives (referring to the referent vs. addressing the referent directly). Her accent-lending rise was analysed as L*H H% or low rise according to Gussenhoven (1984, 2002, 2004). The accent L*H is associated with the meaning ‘testing’ in Gussenhoven’s model. Chen et al. (2014) used three of Casper’s situational contexts for the meaning ‘testing’ to elicit the proper name *Mi* in L*H H% from the default perspective. Their pilot experiment with three native speakers of Dutch confirmed that these contexts could indeed consistently elicit this low-rise contour.

**Figure 2:**
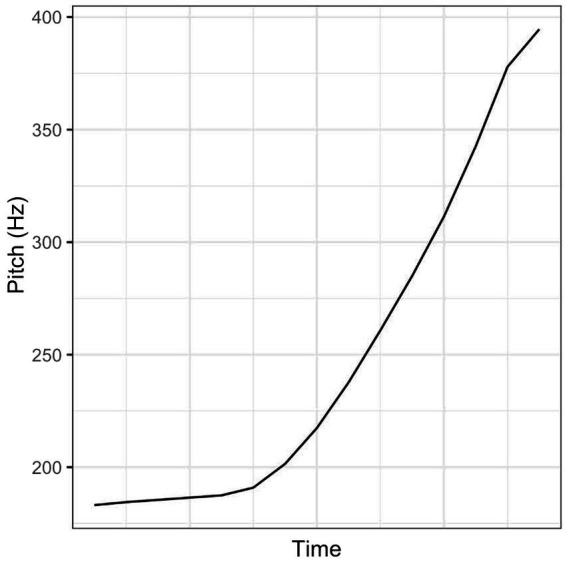
The prototype of L*H in the Dutch one-word utterance Mi.

For the current purpose, we tested the prototypicality of instances of L*H (followed by H%) realised on Mi with or without the original context in a perception experiment. In this experiment, native speakers of Dutch (*n* = 5, 5 females, mean age: 24;8, SD = 3;0) listened to 210 instances of Mi spoken with L*H in three situational contexts by seven speakers, half of them in isolation and the other half in the original situational context, intersected by instances of Mi spoken with H*L (followed by L%), and rated how good the production of the rising pattern was. Prior to the experiment, they were told that the researchers would like to find out what a typical Dutch rising pattern should sound like. They conducted the rating on a 7-point equal-appearing interval scale from ‘bad production of a rising intonation’ to ‘good production of a rising intonation’ using a computer program which allowed them to listen to each recording up to three times. The participants were moderately consistent in their ratings (Cronbach’s α = 0.59), but they all rated the instance of L*H that was the overall favourite highly (≥ 6). The instances of L*H were on the average slightly but statistically significantly higher rated when presented with the context than without the context (mean = 5.23, SD = 1.733 in the context condition; mean = 4.84, SD = 1.886 in the no-context condition; t = 4.419, *df* = 524, two-tailed: *p* < 0.001). However, the instance that was rated the highest (mean = 6.44) in the isolation condition was also rated the highest in the context condition (mean = 6.44). This instance of L*H was then selected as the prototype of L*H (Figure 2). It was produced in Caspers’s (2000) ‘default testing’ context D1B:

Je neemt deel aan een docentenvergadering. Er moet een leerling worden benoemd in het schoolbestuur. Een aantal kandidaten wordt geopperd door je collega’s en je hebt zelf iemand in gedachten waarvan je absoluut niet weet hoe die persoon zal vallen bij de rest; je doet een voorzichtige suggestie: Mi.

[You are attending a staff meeting. A pupil has to be appointed to the school administration. A number of candidates are put forward by your colleagues and you yourself have someone in mind of whom you are absolutely unsure whether that person will be acceptable to the others; you offer a tentative suggestion:]

**Figure 3:**
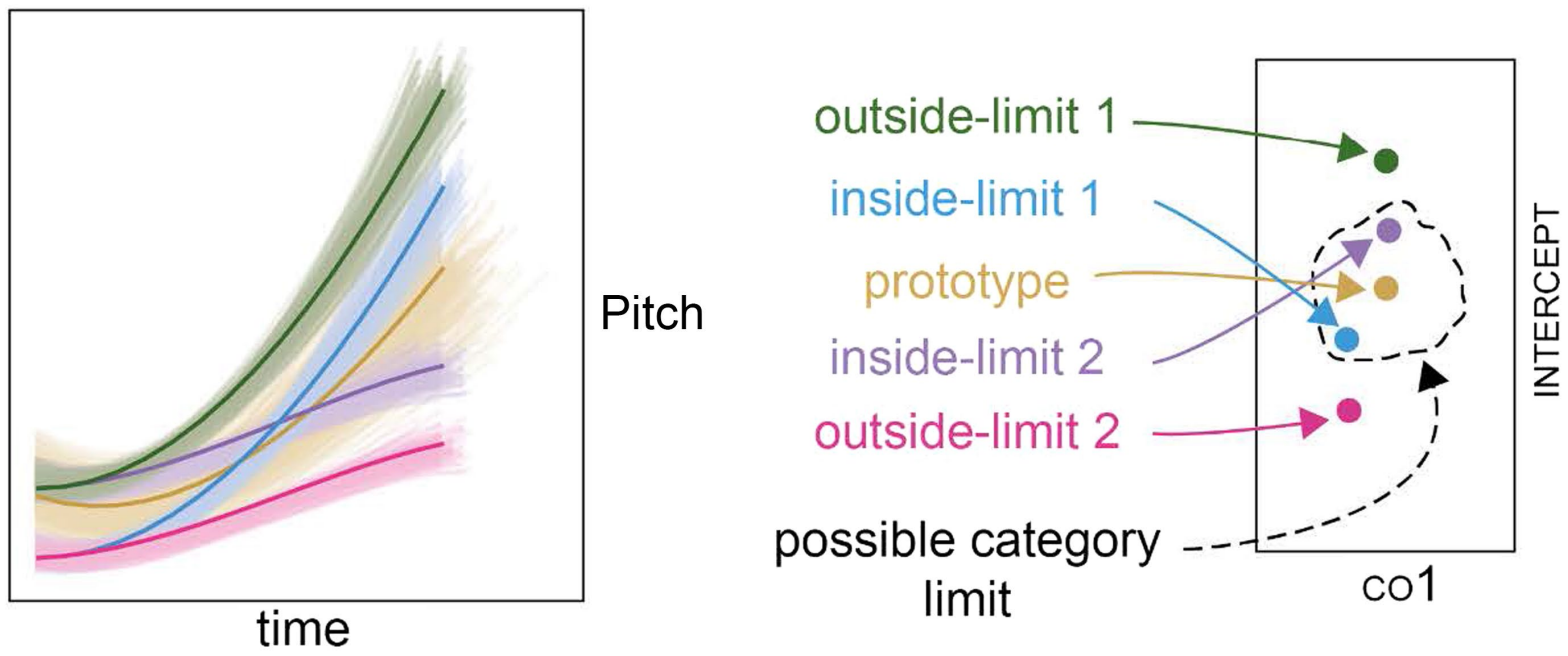
The contour shapes for the referents and neighboring exemplars; gold-prototype referent and the corresponding prototype referent set, blue and purple-two inside-limit non-prototype referents and the corresponding non-prototype referent sets; green and pink-two outside-limit non-prototype referents and the corresponding non-prototype referent sets.

Note that according to Gussenhoven (2004, p. 299) ‘… it (is) hard to discern any meaning difference between the high rise (H* H%) and the low rise’. This suggests that the pitch accent of the rising patterns elicited in Caspers’s (2000) D1A context could also be H* based on the meaning it was supposed to convey. However, Gussenhoven’s (2004, p. 288) description of how the high rise and the low rise should be realised in monosyllabic words and our close inspection of examples of the low rise in the online course on ToDI (Gussenhoven, 2005) suggest that the shape of the rising pattern in our selected instance (Figure 2; and the resynthesized instances of the prototype, see the gold-colored patterns in Figure 3) is comparable to that of L*H followed by H%, not comparable to that of H* followed by H% or any other rising nuclear contours (e.g., L*H %, H* %).

#### Step 2: Extraction of contours and quantifying natural productions using SOCoPaSul

The ProsodyPro Praat script (Xu, 2013) was used to extract the time-normalized pitch contour of each of the tokens in Chen et al.’s (2014) dataset. The SOCoPaSul polynomial model was then fitted to each normalized curve. This gave a cloud of values from naturally produced tokens of L*H for all five SOCoPaSul dimensions. The deviation around the prototype was calculated for each of the dimensions of these tokens.

#### Step 3: Selecting non-prototype referents

From near the edge of the cloud of natural productions of L*H, we selected two points in space to serve as potential non-prototype referents (i.e., the inside-limit non-prototype referents). They were placed at different points in the co1-co2 plane, but at the same distance from the prototype. The other parameters were kept constant at 0. These two inside-limit non-prototype referents are shown as the blue and purple contours in Figure 3.

We additionally created two further referent points out of the range of the natural productions of L*H (i.e., the outside-limit non-prototype referents, see the green and pink contours in Figure 3) by alternating the intercept to give the pink referent the same pitch register as the blue set, and the green referent the same register as the purple referent. The green and pink referents therefore differed from the prototype by the same amount as the blue and purple referents in co1-co2-co3 space, but were further in co1–co2–co3-intercept space. This allowed the testing of the impact of the inclusion of the intercept, and the testing of the impact of different pitch registers whilst holding the size of the excursion and the valley alignment constant. The pitch register was defined as the mean pitch of the first three “time-points” of the contour. The time points were 15 equally spaced points in the temporal dimension, which defined the pitch for the purposes of the manipulation. So in an item with a longer duration, the first three time-points were slightly longer than in an item with a shorter duration. Defining the absolute register in this way was a deliberate choice, because it meant that items that were identical other than their duration scaling received the same value for pitch register. The excursion size was defined as the difference between the highest pitch in the contour and the pitch register. The valley alignment was defined as the number of time points in which the curve that remained within 15 Hz of the pitch at the first time-point.

#### Step 4: Creating neighboring exemplars for each referent

Around each referent, we created a pattern of “neighboring” points (shown as the blurred contours centering each contour in the left panel of Figure 3), arranged in a star-burst pattern, so that there were neighbors that were close to the referent, and neighbors that were further from the referent. Two differently-sized star-burst patterns were defined. The values of each parameter in each star-burst pattern were defined in z-scores. The origin was at point (0,0,0,0,0). In the large start-burst pattern, the first orbit had a radius of 0.3 standard deviations from the referent, the second orbit a radius of 0.6, the third 0.9, the fourth 1.2, the fifth 1.5 and the outermost orbit had a radius of 1.8 standard deviations. The smaller star-burst pattern consisted of the two inner-most orbits of the larger star-burst pattern, those with radii of 0.3 and 0.6 standard deviations. Each point in the five-dimensional space represented a stimulus. Its coordinates represented the parameters that describe it: (intercept, co1, co2, co3, duration). We used the smaller star-burst pattern to create two orbits of neighbors around the prototype referent (used in Experiments 2 and 3) and the larger star-burst pattern to create six orbits of neighbors around the prototype referent (used in Experiment 1) and around each of the non-prototype referent (used in Experiments 1, 2 and 3).

#### Step 5: Resynthesizing the prototype

Target pitch levels for each time-point for each stimulus were calculated using R Statistical Software (R Core Team, 2015) by applying the SOCoPaSul parameters (the coefficients and the intercept) to the polynomial function. Then, gating criteria were applied to ensure that all the synthesized pitch contours would be interpreted as L*H accents. The criteria were that there must be a low plateau of at least 40 ms at the beginning of the contour, where the maximum rise during the plateau was 8 Hz. These criteria were arrived at through informal investigation of the relevant just noticeable differences in conducting ToDI annotation (Gussenhoven, 2005).

After the entire process, the prototype neighborhood created using the bigger star-burst (hereafter the prototype referent set) contained 967 items (see the gold-colored blurred contours in the left panel of Figure 3). Each of the non-prototype neighborhoods (hereafter the inside-limit or outside-limit non-prototype referent sets; see the blue, purple, green and pink-colored blurred contours in the left panel Figure 3) and the prototype neighborhood created using the smaller star-burst (hereafter the near prototype referent set) contained approximately 250 items. A random sample of 150 items was made from each set. Each individual stimulus was created by re-synthesizing the prototype *via* a scripted process, using PSOLA implementation in Praat (Boersma and Weenink, 2012). This gave a separate sound file for each stimulus that consisted of the prototype with the pitch replaced with a curve described by the SOCoPaSul parameters. The inputs to the script to create each individual stimulus were the pitch contours calculated in step 4, and the degree of duration difference between the prototype and the stimulus was calculated.

## Experiment 1: Goodness rating of resynthesized stimuli

To test for the ‘gradient goodness’ symptom of the perceptual magnet effect, a goodness rating experiment was conducted. Participants listened to the stimuli over headphones, and gave ratings on a five point equal-appearing interval scale from ‘bad example’ to ‘good example’ of Mi spoken with a rising melody.

### Participants and materials

Ten native speakers of Dutch (6 females, mean age: 22;2) took part in this experiment. They were students at Utrecht University at the time of testing. All participants rated the prototype referent set. They each additionally rated one of the non-prototype referent sets (two participants each for the inside-limit sets, three participants each for the outside-limit sets). This meant that each participant rated 1,117 items in total.

### Procedure

The experiment was conducted in a web browser using the jsPsych library (de Leeuw, 2015) and the Django Python web application framework (Holovaty and Jacob Kaplan, 2009). It took place in a quiet classroom equipped as a language lab with computers and good quality headphones.

**Figure 4:**
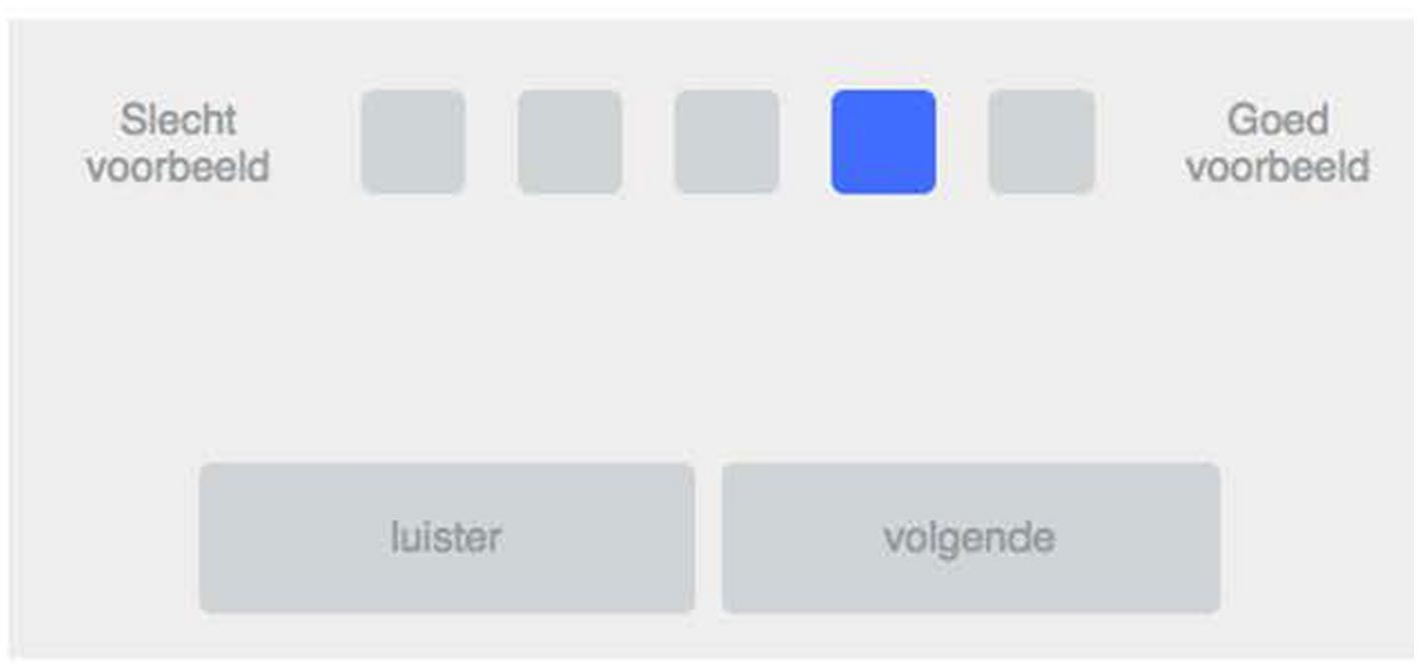
The interface used by the participants to input their ratings, with a rating of four selected, but not yet submitted.

The participants were instructed by means of a slide presentation (also implemented in a web-browser) that they read under the supervision of the experimenter. The key instruction was “determine how typical the rising melody of each example sounds in Dutch.” After the participants were instructed, they did six practice trials to familiarize themselves with the experimental task. The practice trials used the prototype, two items from near the prototype and two items from far from the prototype to familiarize the participants with the extent of variation in the dataset. The presentation order of the items assigned to each participant were randomized by the computer, meaning items from the prototype referent set and from the other non-prototype referent sets were mixed together. Each trial began with the presentation of one item over the headphones. The participants used the mouse to select a rating on a five-point equal-appearing-interval scale, with labels ‘slecht voorbeeld’ (bad example) and ‘goed voorbeeld’ (good example). They could click multiple times to adjust their evaluation if they wished, and listen up to twice additionally to the stimulus by clicking the ‘luister’ (listen) button. When they were happy with the evaluation they had assigned, they clicked, the volgende, (next) button to proceed to the next trial. The interface used is depicted in Figure 4.

The participants completed the rating task in one 1-h appointment and one 40-min appointment on sequential days. The task was broken into blocks of approximately 20 min, with three blocks on the first appointment, and two blocks on the second. There was a mandatory 5 minute break between blocks. After the final block, the participants performed an unrelated task for another study.

### Statistical analysis and results

To prepare data for analysis, each participant’s scores were *z*-normalized, removing variation caused by different participants using subtly different anchors in their scales. This resulted in ratings that vary around 0 (the mean of a participant’s scores), with positive evaluations being rated above 0 and negative evaluations below. The ratings given by the participants in the prototype referent set were moderately consistent (standardized Cronbach’s *α* = 0.59).

To test for gradient goodness, a mixed-effects linear regression model was first fitted using R Statistical Software (R Core Team, 2015) and the package lme4 (Bates et al., 2015) that predicted the mean normalized rating awarded to items in the prototype referent set by distance constructed as the Euclidean distance from the prototype to the item in (co1, co2, co3, intercept, and duration) space. Additional models were built to find out whether the fully specified model could be improved upon by removing some parameters from the calculation of the Euclidean distance. This was done *via* an “all-subsets” approach, by which all plausible models were constructed and then evaluated. Besides, we constructed and tested models using the “naturalistic” metrics typically used in the literature to characterize phonetic realization of pitch accents, i.e., the pitch register, the excursion size, the valley alignment and the duration.

We found that none of the “naturalistic” models using the conventional metrics of pitch accent variation account for the variation in rating as successfully as the best of the models incorporating the SOCoPaSul parameters (Supplementary Table 1), confirming our choice of using parametric modeling to quantify variations in the realization of a pitch accent. Notably, the model that excluded co3 outperformed the fully-specified model. As is depicted in Figure 1, the parameter co3 is the degree of influence that the cubic function contributes to the overall shape. Since the cubic function is sinusoidal, this parameter can be considered to control the degree of deviation from the overall curve at the extremities of the contour, adjusting the flatness of the plateaus at each end of the pitch accent.

**Figure 5:**
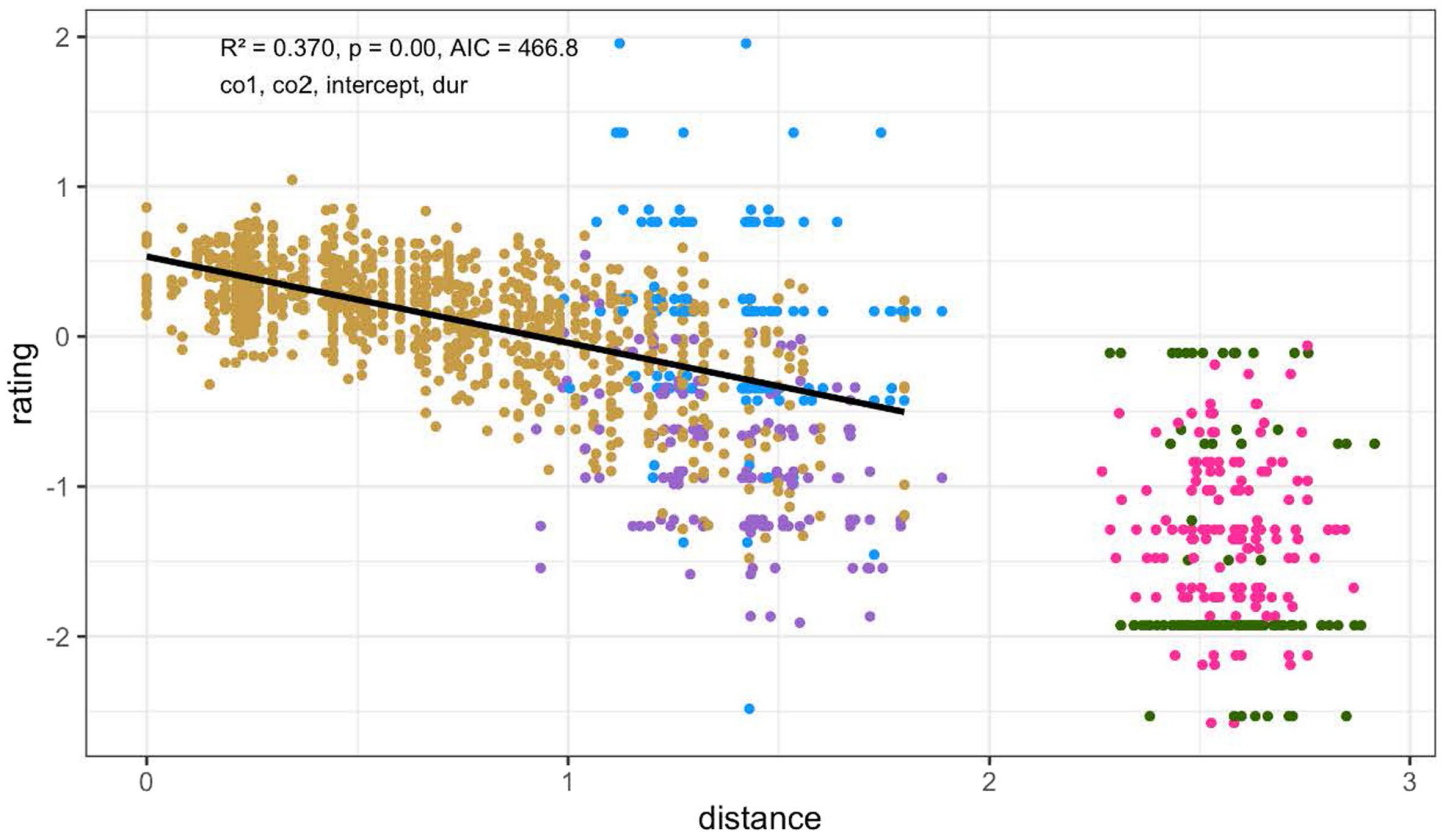
The model that explains the most variation characterizes the distance from the prototype in (co1, co2, intercept, and duration) space. The model is fitted on the ‘goodness’ dataset only, which is colored gold. The other colors represent the ratings on the non-prototype referents and neighboring exemplars. The *x*-axis depicts the distance between a rendition of L*H and the prototype of L*H (the ‘0’ point). The *y*-axis shows the *z*-normalized scores of the goodness ratings, with 0 being the mean of a rater’s scores, positive evaluations being rated above 0 and negative evaluations below 0.

Both the fully specified model (*r* = −0.585, *p* < 0.01) and the best-fitting model (*r* = −0.609, *p* < 0.01) clearly indicated a negative relationship between the distance of a token to the prototype and the goodness rating. The best-fitting model is depicted in Figure 5 and further reported in Table 1. As can be seen in Figure 5, the items closer to the prototype received significantly higher ratings than those further from it and the items from the prototype referent set received by and large the highest ratings, providing evidence for the gradient goodness symptom of the perceptual magnet effect.

**Table 1:** Summary of the best-fitting model for the goodness ratings of the prototype and its referent set.

	Estimate	Std. Error	*t* Value	Pr(>|t|)
(Intercept)	0.533	0.019	28.121	< 0.01
Euclidean distance (co1, co2, intercept, duration)	−0.576	0.024	−23.844	< 0.01

## Experiment 2: Discrimination

An AB discrimination paradigm was employed to test for differential discrimination, presenting the tokens in pairs and asking participants to assess whether or not they heard a difference, similar to Fivela (2012) and Schneider and Möbius (2005). The aim of the task was to establish whether participants were able to detect the difference between a reference sound and a comparison sound.

### Participants and materials

Fifteen native speakers of Dutch (12 females, mean: 21;11) took part in this experiment. They were students at Utrecht University at the time of testing and did not take part in Experiment 1.

All participants were tested on the near prototype referent set and on one of the four non-prototype referent sets in a single session.^2^ As described in “The stimuli,” each set contained 150 items, which were a random sample (the same for all participants assigned to that set) from the 250 items in each referent set. Thus, for each participant, there were 300 test trials (where the comparison sound differed from the reference sound). In the experimental trials, the reference sound in each stimulus pair was either the prototype referent or the non-prototype referent, and the comparison sound was a neighboring exemplar of these referents. In addition, for each type of reference sound, 30 control trials, where the reference sound was played twice, were included to assess the probability of false positives. Each participant was therefore tested on 360 trials. In each set, 75 of the 150 test trials used items taken from the first orbit of the star-burst pattern, 75 used items taken from the second orbit of the star-burst pattern. This means that, besides the control items where there was no difference between the reference sound and comparison sound, there were two levels of difference: small difference (the first orbit) and moderate difference (the second orbit).

### Procedure

The experiment took place in the same quiet classroom setting as Experiment 1, equipped as a language lab with computers and good quality headphones.

The participants were divided into four groups, each of which were tested using a different non-prototype referent set. There were four participants in all groups except that tested with the green-colored outside-limit non-prototype referent set in Figure 3, which had three participants. Each participant was tested on pairs of items consisting of: (1) the reference sound and a comparison sound taken from the neighbors closest to the referent (small difference test trials, 41%), or (2) the reference sound and a comparison sound taken from the neighbors slightly further from the referent (moderate difference test trials, 41%) or (3) the reference sound repeated (control trials, 18%). Two blocks were conducted, one where the reference sound was the prototype (180 trials) and one where the reference sound was one of the four non-prototypes (180 trials). Block order and presentation order within each block were counterbalanced. Whether the target item appeared before the reference sound (AB order) or after the reference sound (BA order) was counterbalanced across participants within each group, so that the items that appeared in AB order for one participant appeared in BA order for the next (and *vice-versa*). The same software packages (jsPsych and Django) were used to implement this experiment as were used in Experiment 1.

**Figure 6:**
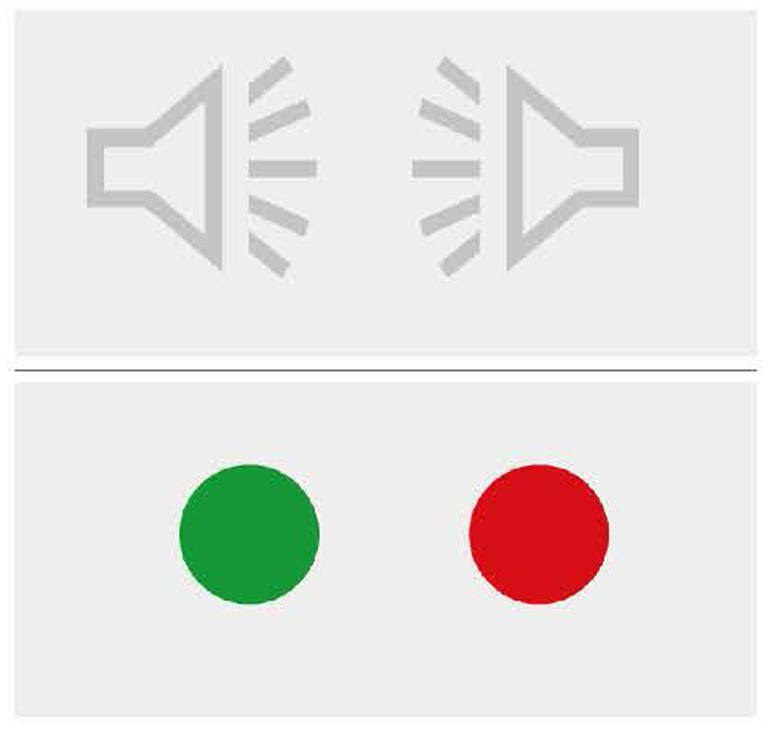
The graphics presented during the discrimination experiment. Upper panel: during playback. Lower panel: signal to respond.

The participants were instructed by means of a slide presentation that they read under the supervision of the experimenter. The key instruction was “determine whether you hear a difference between the two examples.” A keyboard was labelled with a red sticker reading nee “no” on the M key, and a green sticker reading ja “yes” on the Z key. These keys were selected to force the participant to use both hands. The participants were instructed to press the “yes” key if they heard a difference, and to press the “no” key if they did not. On screen, whilst the two sounds were presented, a graphic of two speakers was presented (upper panel in Figure 6). After the offset of the second sound, this was replaced with a depiction of the green and red buttons, as signal to respond (lower panel in Figure 6).

Six practice trials were conducted under the supervision of the experimenter, using items from the prototype referent set that were not selected in the sample of experimental and control trials. These trials represented two control trials, two test trials from the “small difference” condition, and two test trials from the “moderate difference” condition.

The participants completed the task in one block of approximately 30 min. After the experiment, the participants performed an unrelated task for another study.

### Statistical analysis and results

#### Generalization

**Figure 7:**
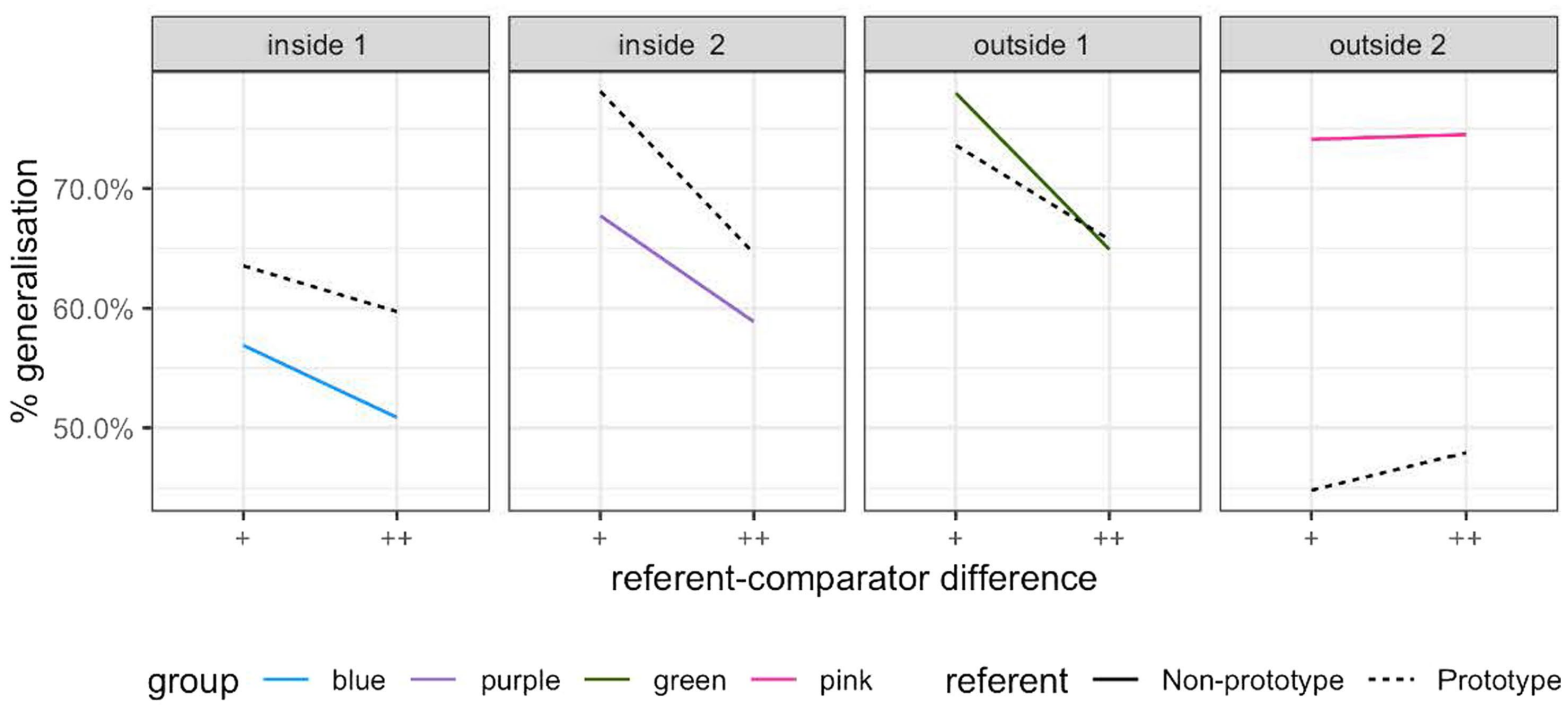
Generalization (misses) in the prototype (dashed) and non-prototype (solid) conditions in Experiment 1.

The participants’ discrimination responses on the test trials were coded as ‘generalized’ if they failed to detect a difference, or ‘not generalized’ if they succeeded in detecting a difference, following Kuhl (1991). As shown in Figure 7, there were more generalized trials in the near prototype condition than in the non-prototype condition in the two groups of participants tested on one of the within-limit non-prototype referent set. But the opposite pattern occurred in the two groups of participants tested on the outside-limit non-prototype referent set: greater generalisation in the non-prototype referent condition than the near prototype referent condition. Furthermore, increasing the difference (from + to ++ in Figure 7) between the comparison sound and the referent sound reduced generalisation for three of the four groups of participants. The participants thus appeared to perform in line with the predictions deriving from the differential discrimination symptom when tested on the within-limit non-prototype referent stimuli in addition to the near prototype referent stimuli. However, the participants differed substantially in their rate of generalisation in the near prototype referent stimuli, which we would expect to be consistent across groups. This implies that there were notable individual differences in the participants’ performance, relative to which non-prototype stimuli were presented to them. This was subsequently confirmed when we plotted the differences between generalisation rates in the near prototype and non-prototype referent conditions, at the participant level (Supplementary Figure 1).

To take individual differences into account, we subsequently conducted mixed-effects binary logistic regression on the whole dataset using R Statistical Software (R Core Team, 2015) and the package lme4 (Bates et al., 2015). The outcome variable was binary (generalized, coded 1, or not generalized, coded 0). The fixed factors were the prototypical status of the reference sound (prototype and non-prototype) and distance of the comparison sound from the reference sound (small and moderate), the random factor was participant nested within group. We began with the random effects model, where only random factors were included. The models with each of the fixed effects on their own, both fixed effects combined, and both fixed effects and their interaction, were tested in a stepwise fashion. After each iteration, fixed effects that did not represent a significant improvement over the previous model (as assessed by comparing the Bayesian information criterion) were excluded. The model with the best fit contained both fixed effects, but excluded their interaction. The criteria used to compare the candidate models are presented in Table 2, and the model with the best fit is shown in Table 3. The model demonstrates that when the trial was in the non-prototype referent condition, there was a significantly smaller chance of generalization, after controlling for variation between participants, than in the near prototype referent condition (*p* < 0.01). For a trial in the near prototype referent condition, moving from the baseline (small difference) in the difficulty dimension to moderate difference (that is, making the trial “easier”) actually increased generalization. This finding was rather unexpected and contra what emerged at the group level (Figure 7) and will be revisited in the “General discussion” section.

**Table 2:** The criteria used to compare the candidate models for the discrimination dataset in Experiment 2.

Fixed factors	Df	AIC	BIC	logLik	Deviance	Chisq	Chi Df	Pr(>Chisq)
(Only random factors)	4	5351.463	5377.111	−2671.732	5343.463	NA	NA	NA
Prototypicality	5	5333.660	5365.720	−2661.830	5323.660	19.803	1	< 0.01
Difficulty	5	5339.117	5371.176	−2664.558	5329.117	0.000	0	
Prototypicality + difficulty	6	5319.654	5358.125	−2653.827	5307.654	21.463	1	< 0.01
Prototypicality * difficulty	7	5321.502	5366.385	−2653.751	5307.502	0.152	1	0.696

**Table 3:** Overview of the best-fitting model for the discrimination dataset in Experiment 2.

	Estimate	Std. Error	*z* Value	Pr(>|z|)
(Intercept)	−0.570	0.243	−2.346	≤ 0.05
Prototypicalitynon-prototype	−0.316	0.068	−4.674	< 0.01
Difficultymoderate difference	0.271	0.068	4.010	< 0.01

#### Response accuracy

Because the result on generalization was coupled with notable differences in performance between the participants, we decided to conduct an explorative analysis on response accuracy in detail. Kuhl (1991) observed in her data that the response accuracy was substantially larger in the non-prototype referent condition than in the near prototype referent condition, in line with the pattern in generalization.

We used the mixed-effects logistic regression modeling technique applied to the generalization data to test for patterns in response accuracy, using R Statistical Software (R Core Team, 2015) and the package lme4 (Bates et al., 2015). In contrast to the models testing generalization, the control trials were included in these models. Therefore, the difficulty factor gained a third level, “no difference,” which became the baseline. The same all-subsets procedure was used to generate and compare models.

The criteria used to compare the candidate models are presented in Table 4. As can be seen, the best fitting model included main effects of the factors prototypicality and difficulty, and the interaction of these two factors, in contrast to the generalization models (Table 5).

**Table 4:** The criteria used to compare the candidate models for the response accuracy dataset in Experiment 2.

Fixed factors	Df	AIC	BIC	logLik	Deviance	Chisq	Chi Df	Pr(>Chisq)
	4	6876.495	6902.872	−3434.248	6868.495	NA	NA	NA
\textsc{prototypicality}	5	6868.264	6901.235	−3429.132	6858.264	10.231	1	< 0.01
\textsc{difficulty}	6	6795.255	6834.820	−3391.627	6783.255	75.010	1	< 0.01
\textsc{prototypicality} + \textsc{difficulty}	7	6782.216	6828.375	−3384.108	6768.216	15.039	1	< 0.01
\textsc{prototypicality} + \textsc{difficulty} + \textsc{prototypicality:difficulty}	9	6778.082	6837.429	−3380.041	6760.082	8.134	2	≤ 0.05

**Table 5:** Summary of the best fitting model for the response accuracy dataset in Experiment 2.

	Estimate	Std. Error	*z* Value	Pr(>|z|)
(Intercept)	0.687	0.172	3.984	< 0.01
Prototypicalitynon-prototype	0.307	0.148	2.069	≤ 0.05
Difficultysmall difference	−1.192	0.119	−10.009	< 0.01
Difficultymoderate difference	−0.969	0.121	−7.973	< 0.01
Prototypicalitynon-prototype:difficultysmall difference	−0.617	0.173	−3.563	< 0.01
Prototypicalitynon-prototype:difficultymoderate difference	−0.572	0.175	−3.278	< 0.01

The main effect for prototypicality was such that the accuracy of trials in the non-prototype referent condition was significantly better than in the near prototype referent condition, in line with the effect of prototypicality on generalization. Difficulty had a surprising main effect: performance was significantly worse in the small difference and moderate difference conditions than in the no-difference condition. Intuitively, an increase in the distance between reference and comparison sound in each stimulus pair should result in improved performance, as the task becomes easier. That this is not the case suggests that the rejection of false positives may be inherently easier than detection of differences. The interactions were also significant; indicating that performance in trials that combined the non-prototype referent condition and the difference-detection task was significantly worse than would be predicted by the main effects alone.

### Interim summary

The results of mixed effects logistic regression for generalization supports the presence of the differential discriminability symptom. In combination with the finding of gradient goodness in Experiment 1, these results indicate a perceptual magnet effect, and therefore evidence that L*H has internal structure. But we also observed notable individual variation in the discrimination of different groups of participants. Because these groups were presented with different sets of non-prototype stimuli, we conducted Experiment 3 using a within-subject design to find out whether such a design could mitigate individual variation in discrimination.

## Experiment 3: Discrimination in a within-subject design

Using stimuli from Experiment 2, we tested 16 native speakers of Dutch (9 female, estimated mean age: 21 ~ 22 years)^3^ for discrimination performance in all four non-prototype conditions. They did not participate in Experiment 1 and Experiment 2 but were otherwise comparable to the participants in the first two experiments.

The stimuli were presented in a blocked fashion. Four blocks consisted of trials where the sounds were taken from the four non-prototype referent sets. Two blocks consisted of trials where the sounds were taken from the near prototype referent set. Presentation order within each block was randomized, and the order of the blocks was pseudo-randomized such that the two identical near prototype referent blocks were separated by at least one other block. Each block took around 13 min, and the participants were obliged to take a three-minute pause and did a small paper-pencil based questionnaire on lexical semantics between each block to minimize participant fatigue and boredom.

In Experiment 2, a computer-equipped classroom was used, with multiple participants being tested simultaneously, using low-specification headphones. Experiment 3 was conducted in sound-isolated booths with high-specification headphones, providing the participants a much less distracting environment. Each testing session lasted about 120 min, including a practice session (see “Procedure” under the section Experiment 2: Discrimination).

### Response accuracy

**Figure 8:**
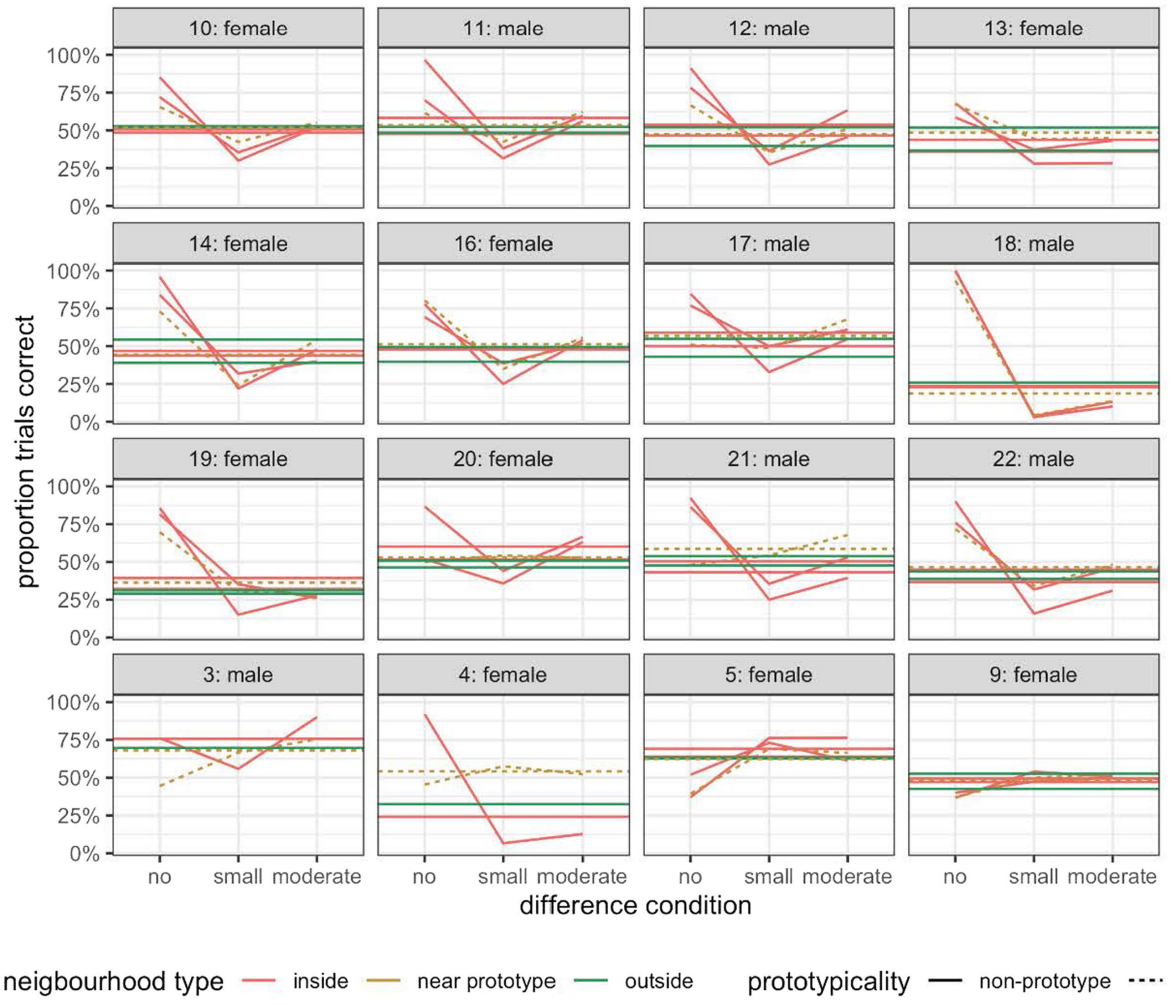
The percentage of trials with a correct response for each participant in Experiment 3.

Figure 8 shows the percentage of trials with a correct response for each participant separately. As can be seen, only participant 18 clearly displayed the differential discrimination symptom of the perceptual magnet effect when we compared the average performance across the three different sorts of trials, i.e., those with no difference, those with a small difference, and those with a moderate difference between the reference sound and the comparison sound.

**Figure 9:**
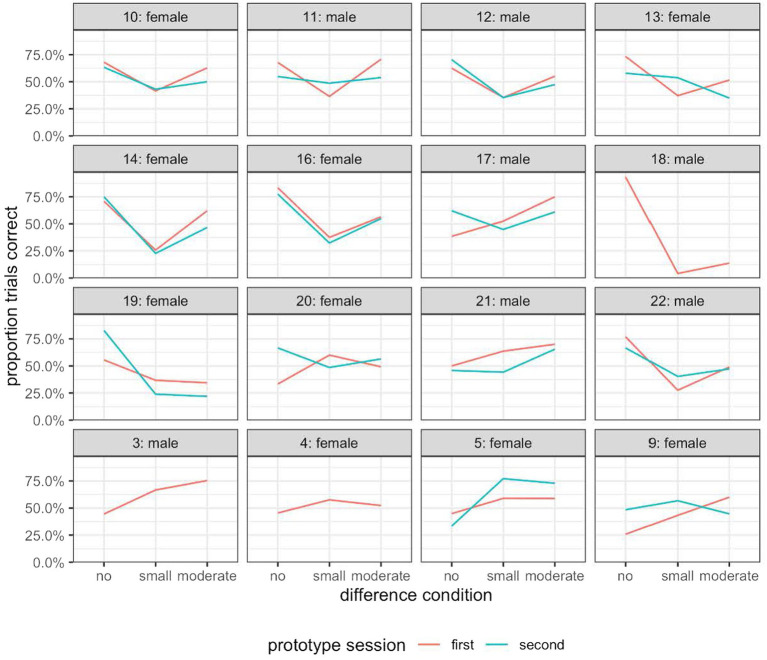
The percentage of trials with a correct response for each participant in the first and second half of the prototype-referent condition in Experiment 3.

The absence of the differential discrimination symptom across the dataset is surprising given the result of Experiment 2. We thus checked for patterns introduced by the methodological changes between this experiment and Experiment 2. Specifically, in this experiment, all but three participants (due to technical problems) rated two blocks of sounds sampled from the near prototype neighborhoods. To rule out a possible training effect, we plotted the participants’ performance for the two blocks of near prototype trials separately. As can be seen in Figure 9, there was a small performance difference between the first near prototype block and the second, but there was no clear pattern of better performance in the second session which would imply a training effect.

To make a more direct comparison with the data from Experiment 2, we plotted the participants’ performance in the near prototype referent condition and inside-limit non-prototype referent conditions, excluding results from the second block of the near prototype referent condition (Supplementary Figure 2). This therefore simulated more closely the task of the participants in Experiment 2. But the results gathered were broadly similar to those obtained with two blocks of stimuli from the near prototype referent set (Figure 9).

### λ-Center as a measure of discrimination performance

Schneider and Möbius (2005) and Schneider et al. (2009) used the λ-center metric to quantify participant success in discrimination tasks, instead of direct accuracy proportions (generalization). The λ-center metric is a concept taken from Signal Detection Theory (Wickens, 2002) and seeks to equalize the performance of different listeners by quantifying the individual response criterion of each listener (i.e., the amount of difference between the two stimuli that the listener requires for them to report a difference). The λ-center is a quantification of that response criterion, using a Gaussian transformation of the proportions of correct hits vs. signal trains and false alarms vs. noise trials. Lower λ-center values indicate better discrimination performance.

**Figure 10:**
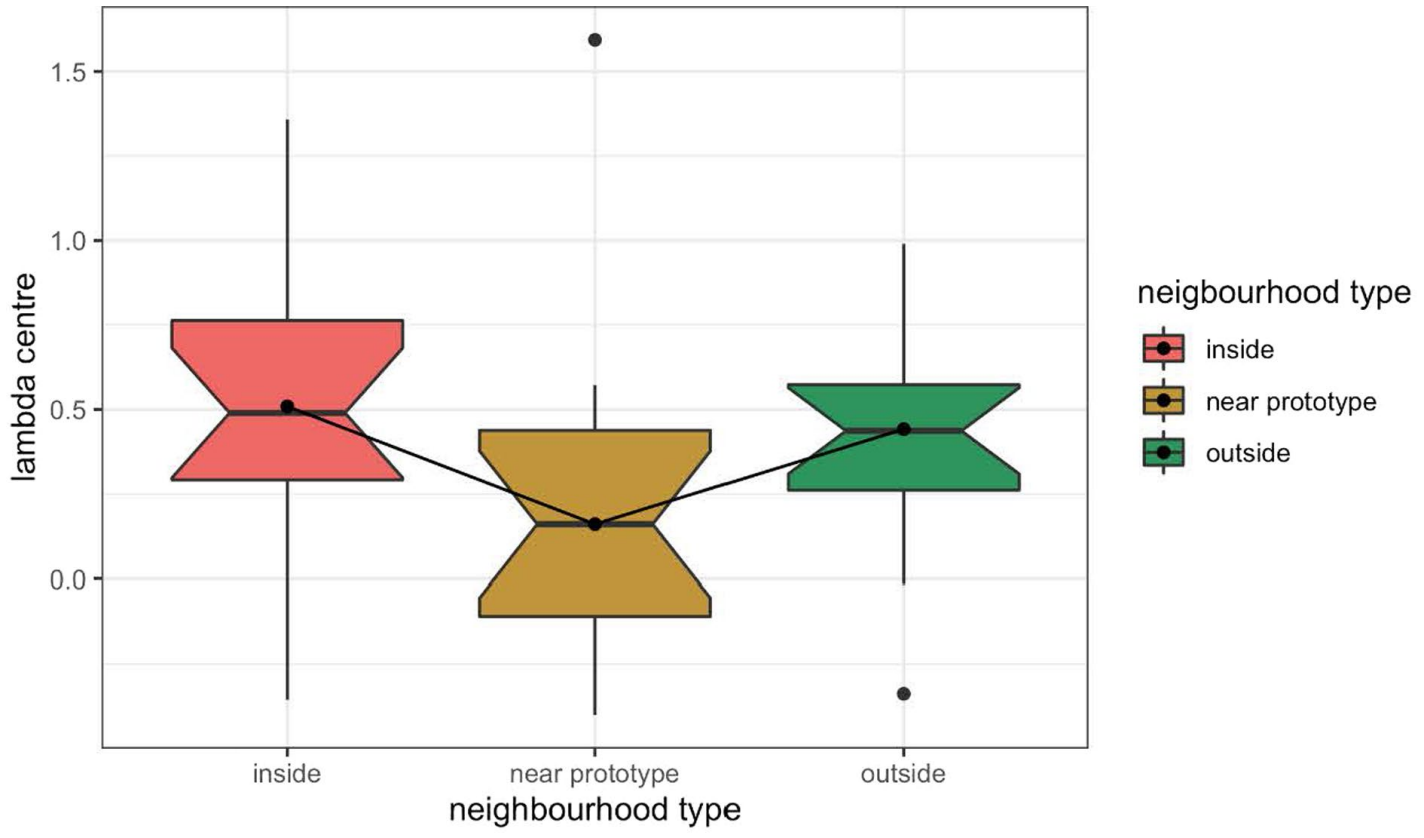
λ-Center for discrimination performance in the prototype referent condition (middle) and in the inside-limit non-prototype referent condition (left) and outside-limit non-prototype referent condition (right) in Experiment 3.

Given that the generalization metric yielded no evidence for the differential discrimination symptom of the perceptual magnet effect in Experiment 3, we decided to analyze the data using the λ-center metric. The λ-center analysis detected better performance near the prototype referent than away from it when averaging across all other factors, against predictions of the perceptual magnet effect, as shown in Figure 10. However, these differences were not statistically significant (inside-limit non-prototype referent set vs. near prototype referent set: *F*(1,29) = 2.57 *p* = 0.1197, outside-limit non-prototype referent set vs. near prototype referent set: *F*(1,29) = 1.62 *p* = 0.2135). When examining the participants’ responses during the first near prototype referent block, the difference between the near prototype referent condition and the non-prototype referent conditions appeared to be slightly more pronounced, with more successful discrimination in the neighborhood of the prototype. Nevertheless, the differences did not reach statistical significance (inside-limit non-prototype referent set vs. prototype: *F*(1,29) = 3.21 *p* = 0.0834, outside-limit non-prototype referent set vs. near prototype referent set: *F*(1,29) = 2.2 *p* = 0.149).

### Interim summary

Unexpectedly, Experiment 3 failed to replicate the results of Experiment 2, in spite of the within-subject design and more favorable acoustical conditions, and arguably more sensitive analysis metric. The approximately equal performance on all conditions suggests that extensive exposure to stimuli with high context variance may influence listeners’ discrimination within an intonational category, different from findings on the within-category discrimination of vowels (Iverson and Kuhl, 2000). We will revisit this finding in “General discussion”.

## General discussion

This study is concerned with the question whether intonational events are speech categories like phonemes and lexical tones. Categoricality of phoneme categories has been experimentally studied by testing discrimination sensitivity peaks at phonemic boundaries (the phoneme boundary effect) and poor discrimination sensitivity within phonemic boundaries, reaching minima near best exemplars of a category (the perceptual magnet effect). In past work, researchers have studied categoricality of pitch accents and boundary tones by examining an intonation boundary effect, the equivalent of the phoneme boundary effect, and to a lesser extent the perceptual magnet effect in the perception of intonational categories. Both lines of research have yielded mixed results. However, animal studies and research on humans using non-speech stimuli have shown that a boundary effect or categorical perception is not necessarily related to categoricality of speech categories. We have thus used improved methodology to examine whether pitch accents have domain-general internal structure of categories by testing the two symptoms of the perceptual magnet effect in the perception of the Dutch L*H pitch accent: gradient goodness and differential discriminability.

Our results of the goodness rating (Experiment 1) demonstrate clearly that the gradient-goodness symptom of the perceptual magnet effect is present in the Dutch L*H pitch accent. The discrimination results of participants tested on the within-limit non-prototypes (Experiment 2) demonstrate that the differential discriminability symptom of the perceptual magnet effect is also present. Thus, the perceptual magnet effect is a feature of the Dutch L*H pitch accent, supporting its postulated categoricality in the phonology of Dutch intonation (Gussenhoven, 2005). This result has both theoretical and methodological implications for the debate on the phonological status of intonation. Theoretically, it suggests that the categoricality of intonational events may not be as controversial an issue as has been perceived on the basis of research examining the intonation boundary effect. Methodologically, it shows the potential of studying the categoricality of other pitch accents and boundary tones by examining the internal structure of the postulated category. Furthermore, that the model using the SOCoPaSul parameters to characterize perceptual distance was more successful than the model using the ‘classic’ quantifications of contour shape variation supports the view that there is merit in such a parametric approach to model intonation contours, and for interpreting the parameters as the dimensions of perceptual space.

However, the evidence gathered appears not to be as strong as that reported for phonemes. For example, the generalization rate was much higher in our study that for the discrimination of vowels (Kuhl, 1991), meaning that it might be considerably more difficult to detect differences between exemplars of pitch accents than exemplars of vowels. Another possibility is that the tone-shift technique used in Kuhl (1991) might be less demanding because it does not require participants to retain the first stimulus in memory for comparison with the second. Furthermore, the general discrimination accuracy was also much lower in this investigation than in Kuhl (1991) (here, in the order of 30–60% correct, rather than the accuracy rates of more than 75% in Kuhl’s study). These differences in the degree of the perceptual magnet effect between intonational events and phonemes suggest that different types of speech categories may differ in the degree of categoricality.

The presence of a main effect for the factor difficulty (or acoustical distance between two stimuli in a pair) in the analysis on generalization in Experiment 2 calls for attention because it is in the opposite direction to that logically expected, i.e., more generalization in the presence of a larger acoustic distance. This finding is difficult to explain. It is perhaps the case that this pattern emerges because of the outside-limit non-prototypes. The participants in one of the outside-limit non-prototypes conditions (the ‘green’ condition) exhibited surprisingly low generalization in the small difference condition and greater generalization in the moderate difference condition. The small sample size and the between-subject design make it impossible to identify reasons why the participants in that group performed differently from the other groups, including the group in the other outside-limit non-prototypes conditions (the ‘pink’ condition). In future research, increasing the number of participants, including checks on factors that can potentially influence pitch perception such as musicality (Schön et al., 2004; Ong et al., 2020) may contribute to a clearer understanding of individual differences in task performance and possibly also mental representation of intonational events.

Experiment 3 was conducted to address the above-mentioned issues using a with-subject design. The result was rather unexpected. Instead of showing stronger evidence for differential discriminability, the participants showed no statistically significant differences in discrimination between the near prototype condition and the non-prototype condition. There are, however, some crucial procedural differences between Experiment 2 and Experiment 3. Namely, in Experiment 3, the participants were presented with many more stimuli (1,080 pairs of stimuli in Experiment 3 vs. 360 pairs of stimuli in Experiment 2) and tested in a much longer session (120 min in Experiment 3 vs. 30 min in Experiment 2) in sound-isolated booths with high-specification headphones. These differences raise the question whether the results were caused by a lack of engagement with the task in the participants. However, in a discrimination task, engagement can also mean that participants attend to subtle differences in a pair of stimuli and manage to discriminate to the same degree across pairs of stimuli, regardless of the acoustic distance between the two stimuli in each pair. This interpretation of participant engagement appears to be in line with the data of Experiment 3. When we only plotted the participants’ performance in the near prototype referent and inside-limit non-prototype referent conditions, simulating the task of the participants in Experiment 2, the results were broadly similar to the results based on the entire stimuli. This suggests that possible participant fatigue-triggered disengagement cannot explain the results of Experiment 3.

Our result may thus imply that very extensive exposure to a large number of exemplars of a hypothetical intonational category coupled with high context variance may influence listeners’ discrimination within an intonational category. This in turn suggests that the state of perceptual magnet effect can be transient as a result of intensive exposure to high context variance. The question arising is whether it is specific to intonational events like pitch accents.

Neural network simulations of distributional learning shows that the transience of the perceptual magnet effect can also occur during the learning of phoneme categories. Using deep Boltzmann machines, Boersma (2019) studied the emergence of phoneme categories as a result of auditory-driven distributional learning of spectral content alone in a simulated first-language learner. He found that the stimulated learner showed a perceptual magnet behavior along a two-dimensional continuum (i.e., F1 and F2 of five vowels) after having listened to 1,000 pieces of data but this behavior faded away as more pieces of data were heard. However, the perceptual magnet behavior seems to be stable and insensitive to the amount of auditory exposure in simulated distributional learning of Mandarin lexical tones. Using the same neutral network simulation,^4^ modeled the distributional learning of four Mandarin Chinese lexical tones on a three-dimensional continuum (onset pitch, medial pitch, offset pitch, pitch contour, sound-meaning mapping). They found that the simulated learner’s perceptual magnet behavior was at its peak after having heard 1,000–1,500 pieces of data, started to decrease afterwards but stabilized after the presentation of 200,000 pieces of data. Together with these findings, findings from Experiment 3 posit a striking difference between pitch accents and phonemes as speech categories on the one hand and lexical tones on the other hand. Future experimental research on the perceptual magnet effects in the perception of lexical tones by native speakers in languages like Mandarin Chinese will be both valuable and necessary in order to attain a clearer understanding of perceptual magnet effects as a feature of tonal categories and the differences between pitch accents and lexical tones. Further research is also needed to tease apart the influence of extensive auditory exposure and high context variance on listeners’ within-category discrimination.

## Limitations

The current study is the first of its kind and inevitably has methodological limitations, which should be taken into account when generalizing its results and in follow-up research. First, the sample size was small, in particular in Experiments 1 and 2. This did not allow a more balanced distribution of male and female participants. Second, the participants listened to a large number of stimuli that were rather similar to each other in Experiments 1 and 3. Although we took measures to mitigate participant fatigue and boredom by inserting short obligatory pauses between blocks of stimuli, it would have been better to have longer breaks and not to conduct unrelated tests during breaks (Experiment 3). Third, the participants were not given a definition of the Dutch rising pattern. Neither were they told that the rise could have either a low or a mid-high start. The participants thus worked with their own notion of rising patterns. In spite of not being given a definition of the low rise under investigation, the participants in Experiments 1 and 2 showed clear evidence that they rated the postulated prototypical tokens of the low rise in the way that we expected based on the perceptual magnet effect. This may in turn suggests that native speakers of Dutch interpret a typical Dutch rising pattern as a low rise. Nevertheless, it would have been recommendable to make clear to the participants what kind of rise they were supposed to listen for by giving examples of Mi in the ‘default testing’ situational contexts. Finally, due to the monosyllabic nature of the stimuli and their being spoken as isolated utterances, the question arises as to whether we have tested the perceptual rating and discrimination of instances of the nuclear contour L*H H%. In line with Gussenhoven’s (2005) description of rises and the boundary tones and Gussenhoven and Rietveld (2000), who used monosyllabic target words in sentence-final position to study the behavior of H* (as in H*L L%) and L* (as in L*H H%), we believe that the variation created in our stimuli does not change the identity of the boundary tone, which is always H% (see Figure 3), but it does change the valley alignment and shape of the rise before it reaches the target of H% and can hence influence the perceived pitch accent category. We thus argue that our results are pertinent to the categoricality of L*H, not that of the entire contour. Nevertheless, future research using multisyllabic stimuli is needed to validate the results of the current study.

## Conclusion

To conclude, our study has put forward the first evidence for the categoricality of the Dutch L*H pitch accent by examining the perceptual magnet effect. This approach shows promise in future research as a means to investigate the categoricality of other pitch accents in Dutch and intonation events in other languages. It is, however, important to take into account that the perceptual magnet effect in perception of intonation may be sensitive to extensive auditory exposure and high context variance.

## Data availability statement

The raw data supporting the conclusions of this article will be made available by the authors, without undue reservation.

## Ethics statement

Ethical review and approval was not required for the study on human participants in accordance with the local legislation and institutional requirements at the time of testing. The patients/participants provided their written informed consent to participate in this study.

## Author contributions

JR and AC designed the study and contributed to the interpretation of the results and the writing of this manuscript. JR conducted the study and analyzed the data. All authors contributed to the article and approved the submitted version.

## Funding

The research was partially funded by a Talent grant awarded to AC by Utrecht University’s strategic theme ‘Dynamics of Youth’.

## Conflict of interest

The authors declare that the research was conducted in the absence of any commercial or financial relationships that could be construed as a potential conflict of interest.

## Publisher’s note

All claims expressed in this article are solely those of the authors and do not necessarily represent those of their affiliated organizations, or those of the publisher, the editors and the reviewers. Any product that may be evaluated in this article, or claim that may be made by its manufacturer, is not guaranteed or endorsed by the publisher.

## Footnotes

^1^Chen et al. (2014) was conducted as part of a larger project on the subcortical processing of pitch in Dutch and Mandarin Chinese. In this type of research, tokens of /mi/ are frequently used as stimuli (e.g., Wong et al., 2007), because it contains only sonorant segments and it is long enough to realize different pitch patterns, including Mandarin lexical tones, but it is not too long for using EEG to track the frequency-following response of the brainstem. For this reason, we used Mi spoken as a proper name with L*H H% and its resynthesized renditions as stimuli in this study.

^2^The prototype-referent set was not used in Experiment 2 because it contained far more items than the non-prototype-referent sets.

^3^Due to loss of information on the participants’ age, we estimated their mean age based on the information on the participants in Experiment 1 and Experiment 2, who were recruited from the same student population and were comparable in academic background and age.

^4^Yang, J. (2020). *Distributional Learning of Mandarin Lexical Tones in Bidirectional Deep Neural Network*. Unpublished report. Amsterdam: University of Amsterdam.
