# Supplementary material for: Internal structure of intonational categories: The (dis)appearance of a perceptual magnet effect
Source: Front Psychol. 2023 Jan 17;13:911349. doi: 10.3389/fpsyg.2022.911349 (PMC9887997; doi:10.3389/fpsyg.2022.911349)
Supplement: Supplementary file 1 [file Table_1.docx]

Supplementary Material

**Supplementary Table 1.** The quality of the fit of various models for the goodness ratings of the prototype and its referent set in Experiment 1.
