# Supplementary figures and images for: Internal structure of intonational categories: The (dis)appearance of a perceptual magnet effect
Source: Front Psychol. 2023 Jan 17;13:911349. doi: 10.3389/fpsyg.2022.911349 (PMC9887997; doi:10.3389/fpsyg.2022.911349)

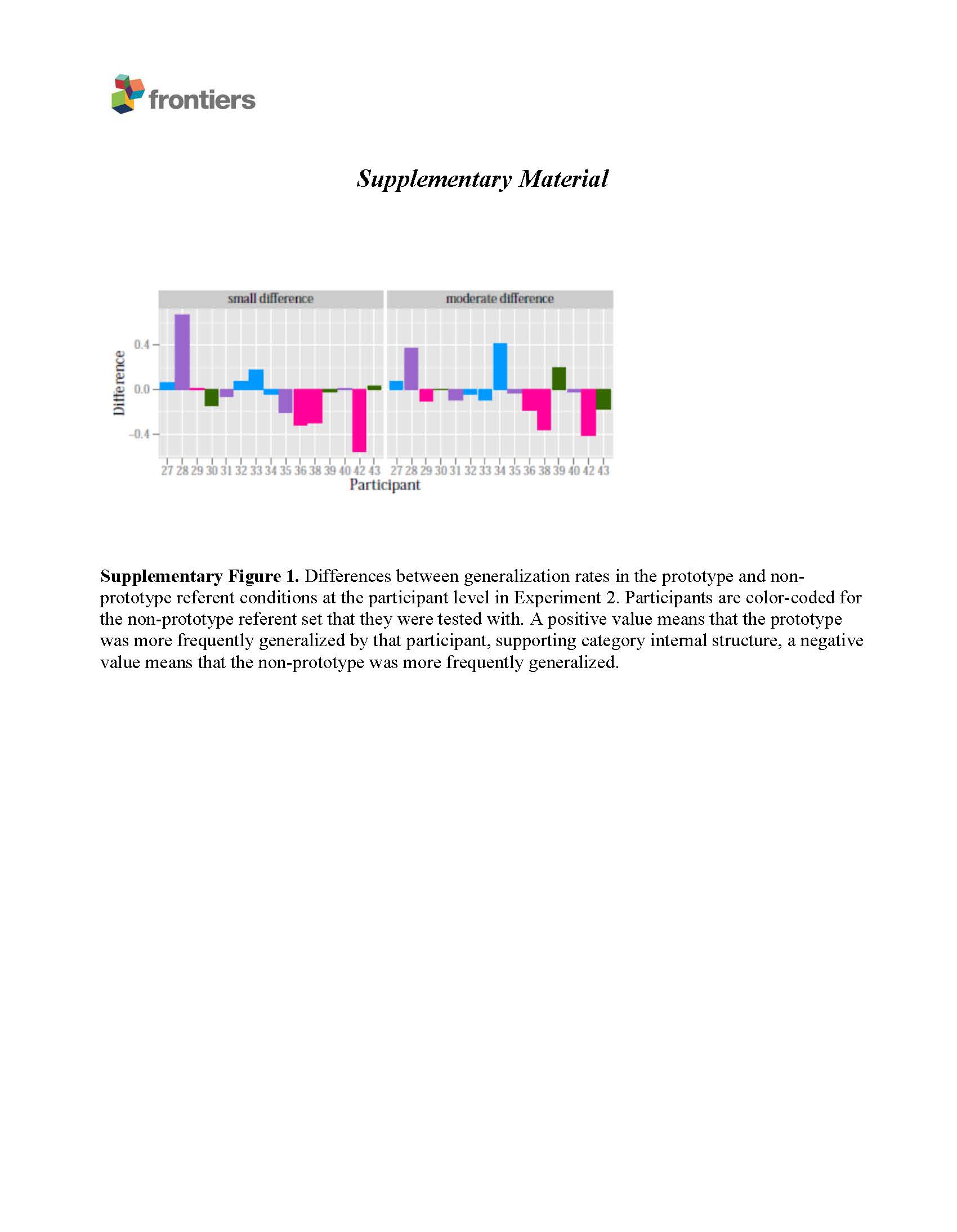

Supplement: Supplementary file 2 [file Image_1.jpg]

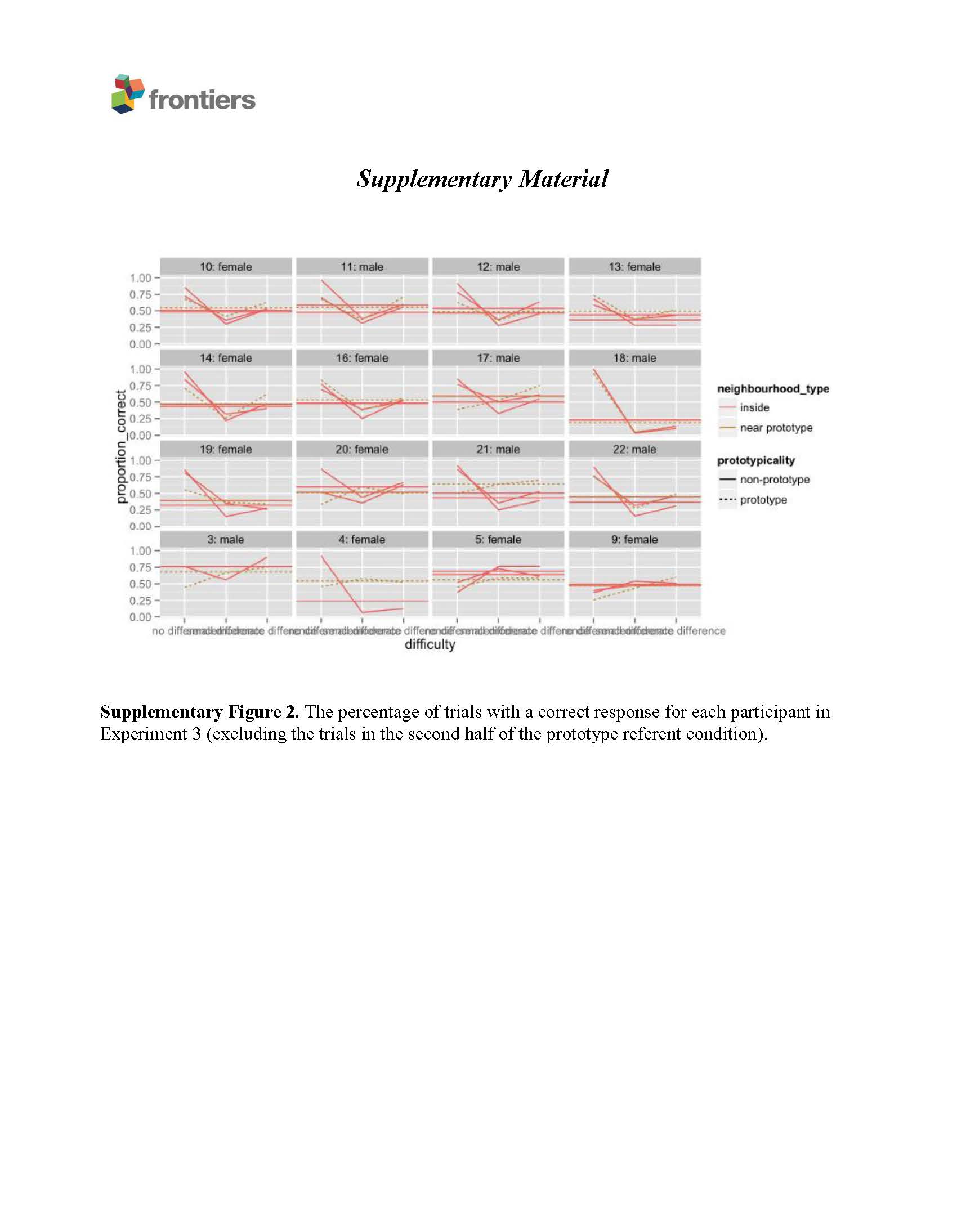

Supplement: Supplementary file 3 [file Image_2.jpg]
